# Medical Needling: Effect on Skin Erythema of Hypertrophic Burn Scars

**DOI:** 10.7759/cureus.3260

**Published:** 2018-09-06

**Authors:** Kay-Hendrik Busch, Antigona Aliu, Nicole Walezko, Matthias Aust

**Affiliations:** 1 Waldkrankenhaus Bonn, Johanniter Kliniken Bonn, Bonn, DEU; 2 Medical Faculty, Heinrich-Heine-University, Bonn, DEU; 3 Anästhesiologie, Marienhospital Bonn, Bonn, DEU; 4 Plastic Surgery, Waldkrankenhaus Bonn, Bonn, DEU

**Keywords:** medical needling, pathological persistent erythema, hypertrophic burn scars, vascularization, epidermal thickness

## Abstract

Background

Burn scars frequently tend to have pathological discolorations, which is manifested in the development of persistent erythema. Affected people suffer from psychological and physiological issues when they are restricted or rejected in their daily life. In this context, medical needling seems to be an efficient therapy for erythematous scars with a relatively low-risk rate of postoperative complications. Study research has already shown significant improvements in the scar quality with reference to the parameters “moisture and transepidermal water loss.” Clinical data is up-to-date and provides an innovative therapy outcome of scar treatment with medical needling.

Objective

The aim of our study was to examine the influence of medical needling on the pathological and persistent erythema of hypertrophic burn scars. By means of reliable measurement methods, we were able to prove positive and sustainable outcomes for normal and healthy skin. The patient cohort included 20 patients with an average age of 34.63 years. Our examinations involved scars that were at least two years old and had healed by secondary intent. Every scar showed the pathological values of persistent erythema according to the participation requirements.

Methods

For the practical implementation of medical needling or percutaneous collagen induction (PCI), we used a roller covered with needles of 3 mm length. The needling device is rolled over the scar alternatively in a vertical, horizontal, and diagonal orientation. Multiple micro-wounds at a close distance cause intradermal bleeding, which evokes modified skin regeneration provoked by the effects of medical needling. Every patient has been followed up for 12 months postoperatively. Further on, valid results have been evaluated objectively as well as subjectively by the patient and observer.

Results

Our study has shown that persistent erythema of hypertrophic scars can be considered as an indication of PCI. The needling procedure influences vascularization by stimulating angiogenesis in the post-needling wound healing cascade. As the method is based on percutaneous collagen induction, the synthesis of collagen improves the vital thickness of the epidermis, which is directly associated with less transparency. Examined scars showed a significant reduction of erythema and were less reddened after treatment. Based on the outcomes of objective measurements, medical needling achieves a normalization of the skin color and an adjustment to healthy skin after repetitive treatments.

Conclusion

Medical needling seems to be a suitable therapy approach for treating erythematous, hypertrophic burn scars.

## Introduction

Burns represent the fourth leading cause of injuries worldwide [1]. A large number of burn victims are frequently confronted with dysfunctional and aesthetic deficits in their daily life. Affected people may suffer from further harm through stigmatization and social stress due to the pathological changes of their scarring, from slightly reddened skin to persistent erythema. Therefore, modern medicine encourages the quest for less invasive but effective medical treatments in terms of normalizing scar conditions towards healthy and vital skin. The past has shown difficulties in treating hypertrophic burn scars, which are characterized by a complex anatomy and a progressive degradation of the scar formation. Rapid and uncontrolled growth, a lack of moisture and flexibility as well as significant discolorations of the skin impede therapy approaches. As a higher density of blood vessels and increased perfusion can be identified in this prominent kind of scar, visible and persistent erythema can be common attendant symptoms [2]. Distinct and persistent changes in skin color are often related to burn scars and categorized as scars with pathological erythema. Deep degree burns destroy the epidermal structure and affect dermal layers containing important skin cells such as keratinocytes and fibroblasts. Widespread and deep damage through burns leads to the formation of scar tissue within the conventional wound healing process. The initial phase of post-inflammation is marked by a vasodilatation and an increased number of blood vessels due to the active processes of angiogenesis. Thus, the locally intensified vascularization favors blood circulation, which enhances the development of erythema. Another important factor is a thinner epidermis of the scar tissue, showing a parallel orientated collagen structure, which is more transparent for local blood flow. This is why a thinned-out skin texture in scars supports the effect of erythema, as the intense circulation of blood becomes more apparent. Increased skin redness is not only considered an inflammatory symptom but also, quite the opposite, erythema underlies the structural modifications of subdermal components such as the vascular system. Hence, the definition of persistent erythema as a pathological parameter shows clinical relevance as patients show obvious differences in an excessive reddened scar compared to the surrounding healthy skin [3].

In this context, our study is aimed to examine whether medical needling improves the appearance of erythema in hypertrophic burn scars. Measurements were performed with the Mexameter (Courage-Khazaka Electronic, Cologne, Germany) in order to determine the manifestation of pathological erythema based on quantitative measurements of the amount of hemoglobin in the vascular system of the affected area. Current medical standards offer various therapy approaches but stick to conventional treatments in most instances. Therapy outcomes are frequently restricted and are not representative of satisfactory solutions. Conservative treatments, such as silicone patches or intralesional cortisone injections, affect erythematous scars in terms of concealing the problem of persistent erythema [4]. The efficiency of the silicone gel is based on a long-term application and utilization [5]. Patients are confronted with a permanent treatment of the scar area and frequently stigmatized due to the application of a striking gel. According to this, the substantial problem of erythema is not solved but rather undermined by the silicone. Compared to that, corticosteroid injections reduce excessive scar growth by inducing less intense collagen synthesis. Clinical experience based on measurements with the ChromaMeter (Konica Minolta Sensing, NJ, US) showed a fairly limited improvement in erythema [6]. Hence, this alternative method is rather suited for proliferating, active scars in terms of a short-term solution. Furthermore, the maximal treating area is limited due to the additional administration of medication, which might cause further psychological stress for the patient. Clinical data also indicate a significant reduction of erythema through laser treatment, which leads to a measurable reduction in the blood circulation and redness of the affected area [7]. Nevertheless, ablative treatments by means of laser resurfacing or dermabrasion hold the risk of persistent erythema by destroying epidermal structures and deeper dermal layers. As a result, an inflammatory response or a degradation of the scar condition can eventually occur [8]. Moreover, further complications are compounded when a postoperative re-epithelialization is not assured due to a lack of skin appendages and stem cells. In that case, the typical effects of a radical ablation of the skin (including scar tissue and healthy skin) make regenerative processes almost impossible. In contrast, medical needling overcomes the deficits of conventional treatments with a focus on sustainability. Needling therapies can be safely repeated after short time periods and are suitable for almost all body areas for which a visible and structural change is desired. The non-ablative method of PCI displays a minimally invasive intervention that does not destroy the epidermis but rather promotes the formation of physiological collagen instead of scar collagen [9]. The needling cascade initiates an effective regeneration of the skin in terms of a dermal reorganization. Histological findings show a post-interventional re-epithelialization and a thickened epidermis, which both come together with a reduction in skin redness. In this study, we aim to prove the effect of medical needling on skin erythema by means of objective and quantitative measurement methods.

## Materials and methods

Study design

We performed a prospective, randomized, controlled study to compare the effects of medical needling on a patient cohort that met the criteria of hypertrophic scars. A certain number of scars showed pathological erythema, which is the target parameter in this study. Therefore, we divided target scar areas into two subareas that were randomly selected for two groups of positive (medical needling) and negative control (no treatment). In order to provide valid and useful data, a control group of untreated scars was involved. Cohort effects were avoided, as we did not perform an initial sample selection of patients before starting the study. An evaluation of therapeutic outcomes regarding erythema was conducted at baseline and within a three, six, and 12-month follow-up examination of treated and untreated scars as well as a healthy reference for comparison. Every treatment with medical needling was followed by postoperative measurements and photo documentation. The final statistical analysis is based on one-year follow-up results in order to allow a uniform assessment and avoid a source of irritation through external factors. Post-interventional outcomes were assessed by both using the photo documentation as well as the patient and observer scar assessments (POSAS). As we did not integrate double-blind techniques in our study, a patient-observer-bias regarding the subjective evaluation of postoperative results cannot be excluded. Objective data was given by measurements with the Mexameter.

Subject selection

Our study involved patients that have mature hypertrophic burn scars healed by secondary intention. Target scars were at least 10 cm^2^ in size, two years post-accident, and showed pathological erythema. The assessment involved every scar with pathological values of the relevant parameters that were measurable at the beginning of the study. The comparison with healthy skin allowed the definition of pathological discolorations departing from normal skin color. Therewith, we were able to determine pathological values either above or under the normal range of the healthy reference. Exclusion criteria were severe diseases, skin lesions like infections and cancer, as well as pregnancy. Furthermore, involved scars were not treated in any other form before or during the study, except for what is instructed for preoperative management before needling.

Procedure

In Germany, medical needling is a licensed therapy for burn scars that is especially integrated with the fields of plastic and reconstructive surgery. The intraoperative procedure includes general anesthesia and PCI itself. Pre- and postoperative examinations were performed in vivo at all times. Study participants were obligated to sign an informed consent form in order to take part in the follow-up examination as well as provide photo documentation. In the case of patients under 18 years of age, we obtained informed consent from their parents. Preoperative management was kept simple and included the application of vitamins A, C, and E as antioxidants. The effectiveness of pre-treatment regarding maximal outcomes has been already researched in the study by Aust et al. [10].

Medical needling

**Figure 1:**
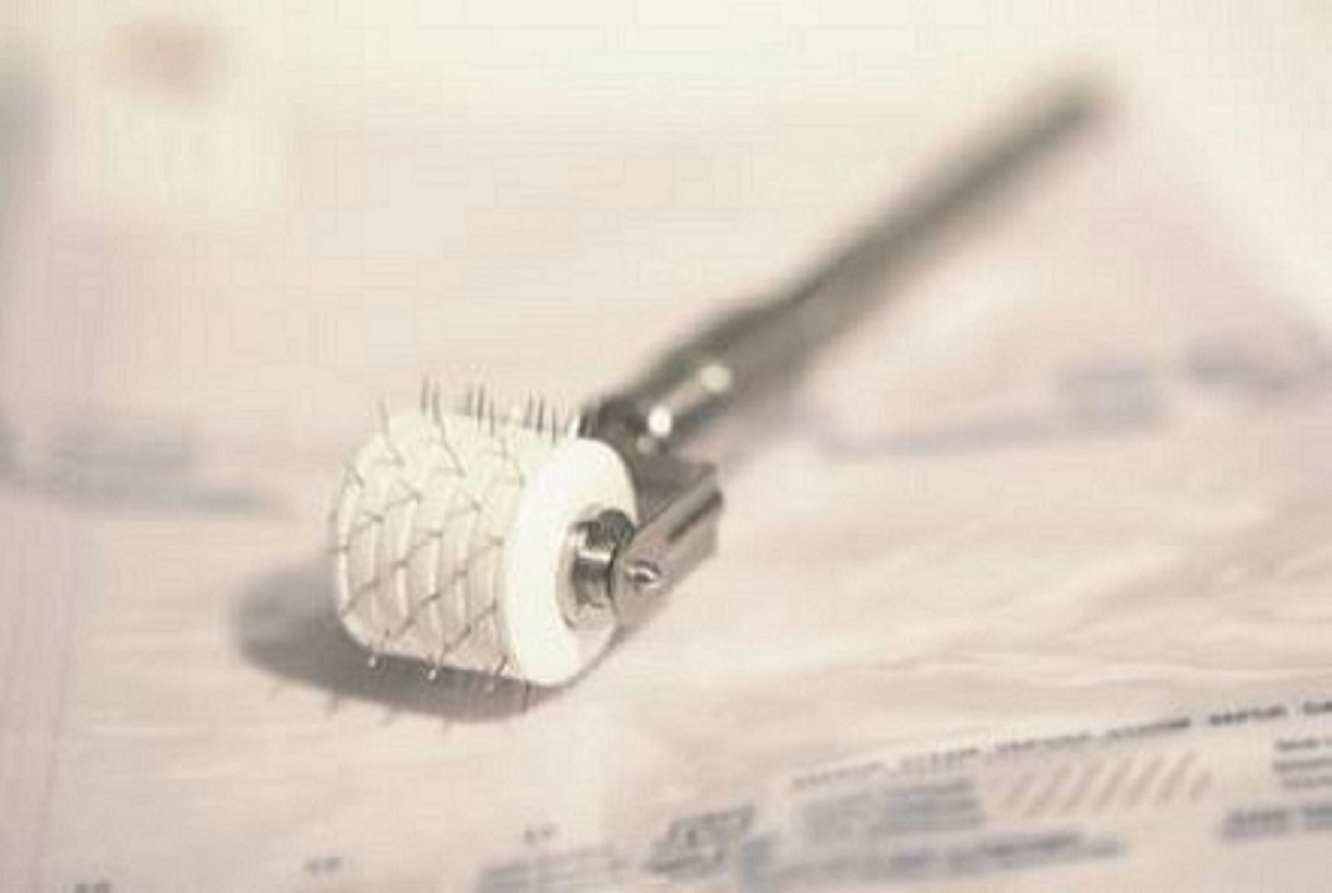
Roller device for medical needling

The needling device is covered with 3-mm long needles that have to be rolled over the scar in three directions under constant pressure: vertically, horizontally and diagonally (Figure 1). A straight guide of the device is important to prevent shear forces and deeper damage. Relative to the extent of the scar, this procedure requires 30-60 minutes of mechanical exposure. The penetration of the papillary dermis leads to thousands of micro-wounds and intradermal bleeding through the parenchymal canals. Minimal lesions of the epidermis do not impair the basal layers containing stem cells with regenerative capacity. The increased expression of specific growth factors and the release of structure proteins induce a modified wound healing cascade with a great regenerative potential. The scar is sufficiently needled when multiple and confluent ecchymoses, as well as skin swellings, are clearly indicated. After 24 hours, epithelial cells have closed the channels and reorganized into a natural protective barrier, which reduces the risk of potential postoperative complications such as infections. Therapy benefits are optimized by the application of nourishing products (vitamins A, C, and E based or tea tree oil-containing cremes) in the first 24 hours. Swelling and local redness of the treated area disappear after approximately four to seven days.

Assessment

**Figure 2:**
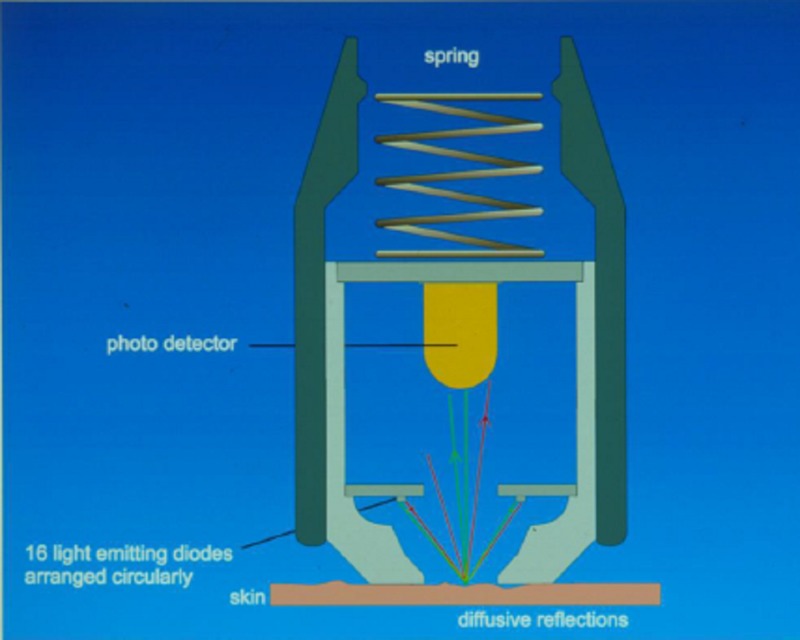
Measurement principle of the Mexameter Image source: Courage-Khazaka GmbH, Cologne, Germany

Subjective evaluation was performed by means of POSAS whereas objective data was provided by using the Mexameter, which quantifies the presence of melanin and hemoglobin [11] (Figure 2). In this study, we concentrated on the in-vivo examination of the amount of hemoglobin, which is closely related to the presence of erythema. POSAS is a patient and observer-administered scale and covers an array of scar characteristics that are equal for both observer and patient. In this study, we concentrated on the evaluation of “color,” “perfusion,” and “overall opinion.” A 10-point ordinal scale was used to draw pre- and postoperative comparisons between the two groups. Further on, each scar was photographed before and after the treatment under uniform conditions of setting, light, and positions of the subject. The hemoglobin measurements are based on the absorption of light. The device’s probe emits light in defined wavelengths, which is 568 nm for hemoglobin. It detects the reflected amount of the emitted light within a 5 mm^2^ skin sample in order to determine the erythema index. Erythema results from a vasodilation due to the formation of the scar, which means increased perfusion in the affected skin area. Reddened skin leads to an absorption of green light, which has the same absorption rate as the oxyhemoglobin of the erythrocytes. Measurements were made three times in a row within a 1 cm^2^ sequence at the same skin location. They included each of the subjects' scars, treated and untreated, as well as healthy skin for comparison.

Statistics

The software SPSS (IBM Corp., Armonk, NY, US) was used for statistical analysis and the program Microsoft Excel for Windows (Microsoft Corporation, Washington, US) enabled the data collection. Further on, the Wilcoxon signed-rank test was implemented due to the limited participation of 20 patients. The statistical significance level was given as p < 0.05.

## Results

Subjects

Twenty subjects, one Asian and 19 Caucasian, were available as the baseline conditions of this study. The patients showed an average age of 34.63 years with a range of 6 to 60 years. The average rate of burned body surface was 32.42% and the average age of the scar at the time of the treatment was 11.42 years. The patient cohort involved 47 scars out of 17 patients with the pathological values of persistent erythema before the treatment with medical needling. The data of 17 examined patients was relevant for the final assessment of the postoperative effect on erythema.

POSAS

Seventeen patients appearing for the follow-up examination were considered for the final assessment. The database involves values from the patient’s last visit (six to 12 months). The follow-up examination did not reveal any infections, as treated scars were 100% epithelialized after that period of time. Subjective data given by POSAS points out an improvement in the scar quality with a significant tendency to normal skin. Each category was equally evaluated by the patient and the observer. Both shared a homogenous evaluation of the skin color that showed an improvement of 43% towards normal and healthy skin.

Patient ratings

**Figure 3:**
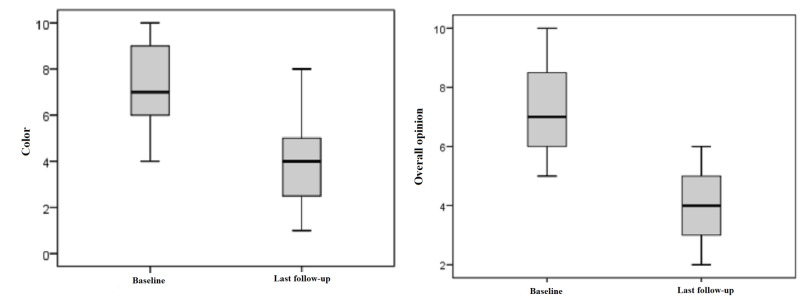
Patient ratings for "color" and "overall opinion" preoperatively and at last follow-up Patient ratings: 1 = as normal skin, 10 = very different from normal skin Box displays lower and upper quartiles, median value is indicated by the horizontal line, endpoints of the upper and lower whisker display minimum and maximum values.

Patients evaluated skin color preoperatively with a mean value of 7.11 points. Postoperatively, they rated 4.05 points at an average, which is an improvement of 50%. Additionally, “overall opinion” regarding scar quality was preoperatively rated with 7.05 points and with 3,95 points at an average, which means an improvement of 51%. This result is statistically significant with p < 0.05 (Figure 3).

Observer ratings

**Figure 4:**
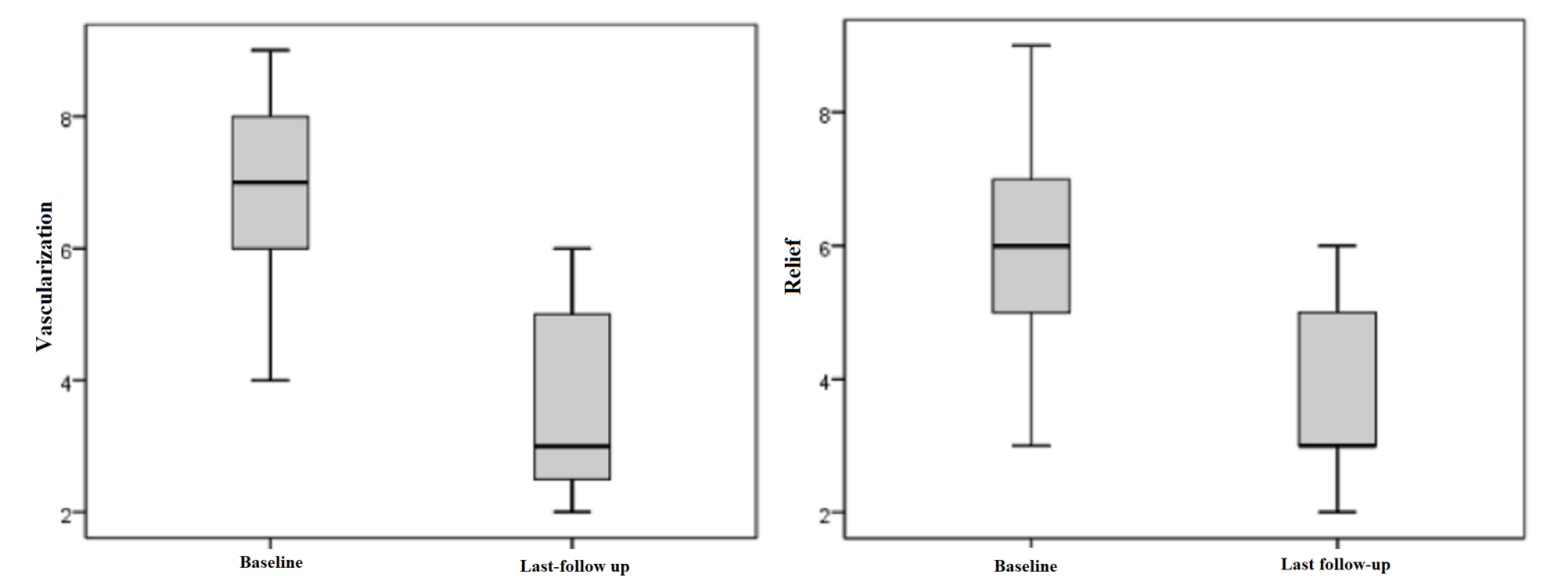
Observer ratings for "vascularization" and "relief" preoperatively and at last follow-up Patient ratings: 1 = as normal skin, 10 = very different from normal skin (Box displays lower and upper quartiles, median value is indicated by the horizontal line, the endpoints of the upper and lower whisker display minimum and maximum values)

The observer scale showed a similar outcome. The parameter “vascularization" was preoperatively rated with a mean value of 6.74 and with 3.58 points postoperatively by the observer. This difference displays an improvement of 55% and is statistically significant with p < 0.05. The observer evaluated the scars with an overall opinion of 6.21 points preoperatively. Postoperatively, they rated this category with 3.53 points on average, which is an adjustment of approximately 47% towards healthy skin. Statistical significance is given as p < 0.05 (Figure 4).

Photo documentation

**Figure 5:**
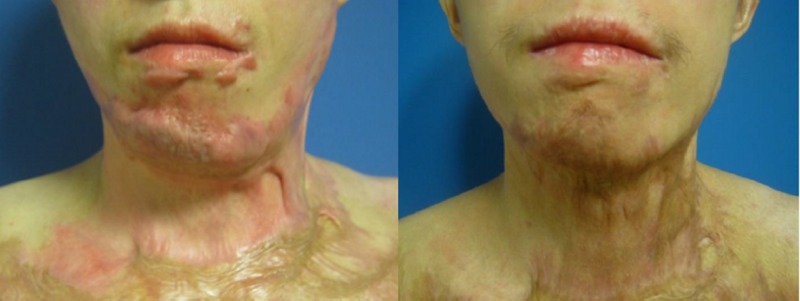
Case 1, frontal shot, preoperatively (left) and one year postoperatively after needling (right). Areas treated: face perilabial, chin, neck

**Figure 6:**
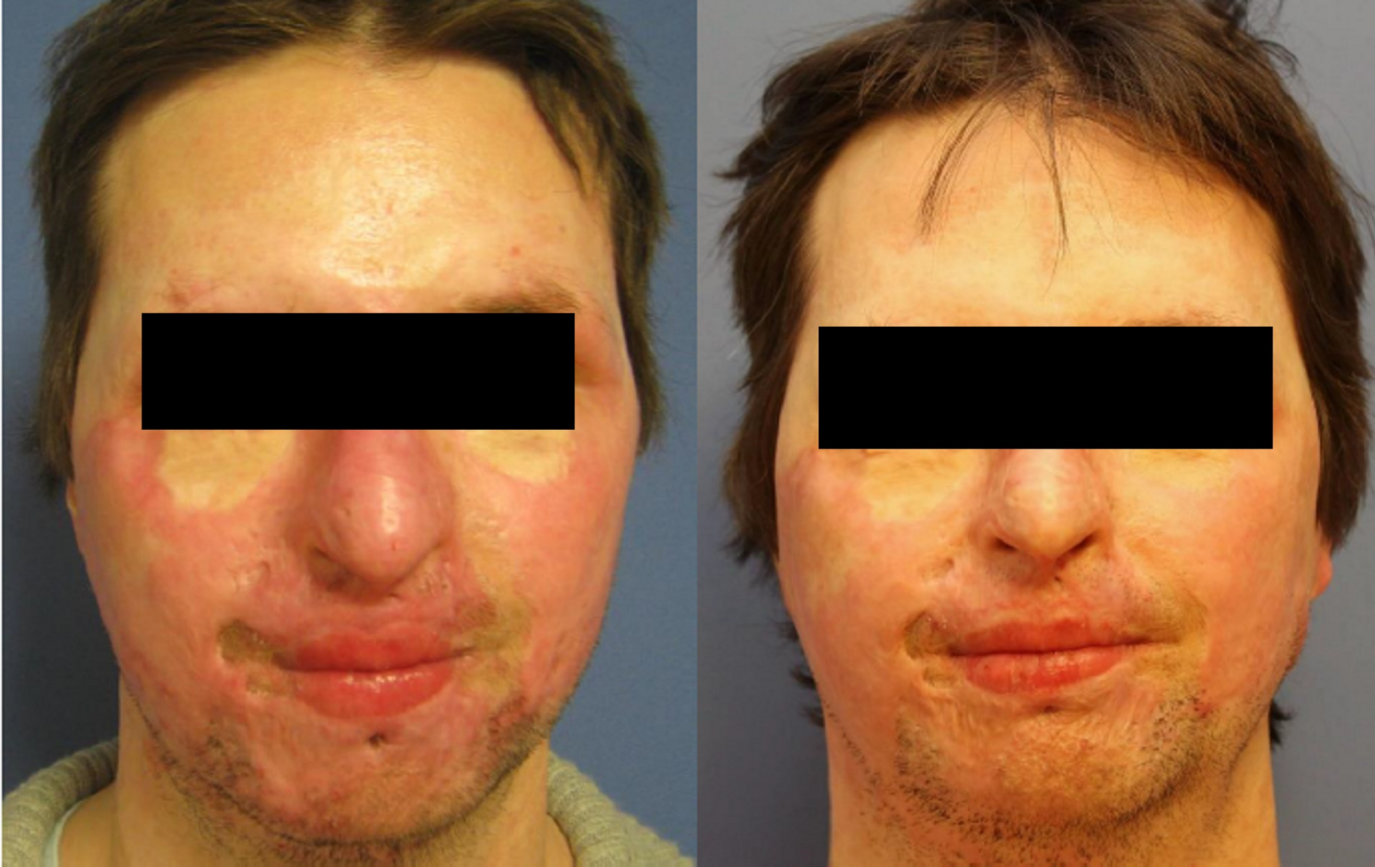
Case 2, frontal shot, preoperatively (left) and one year postoperatively after needling (right). Areas treated: entire face

Exemplary outcomes are now shown (Figures 5-6).

Mexameter

**Figure 7:**
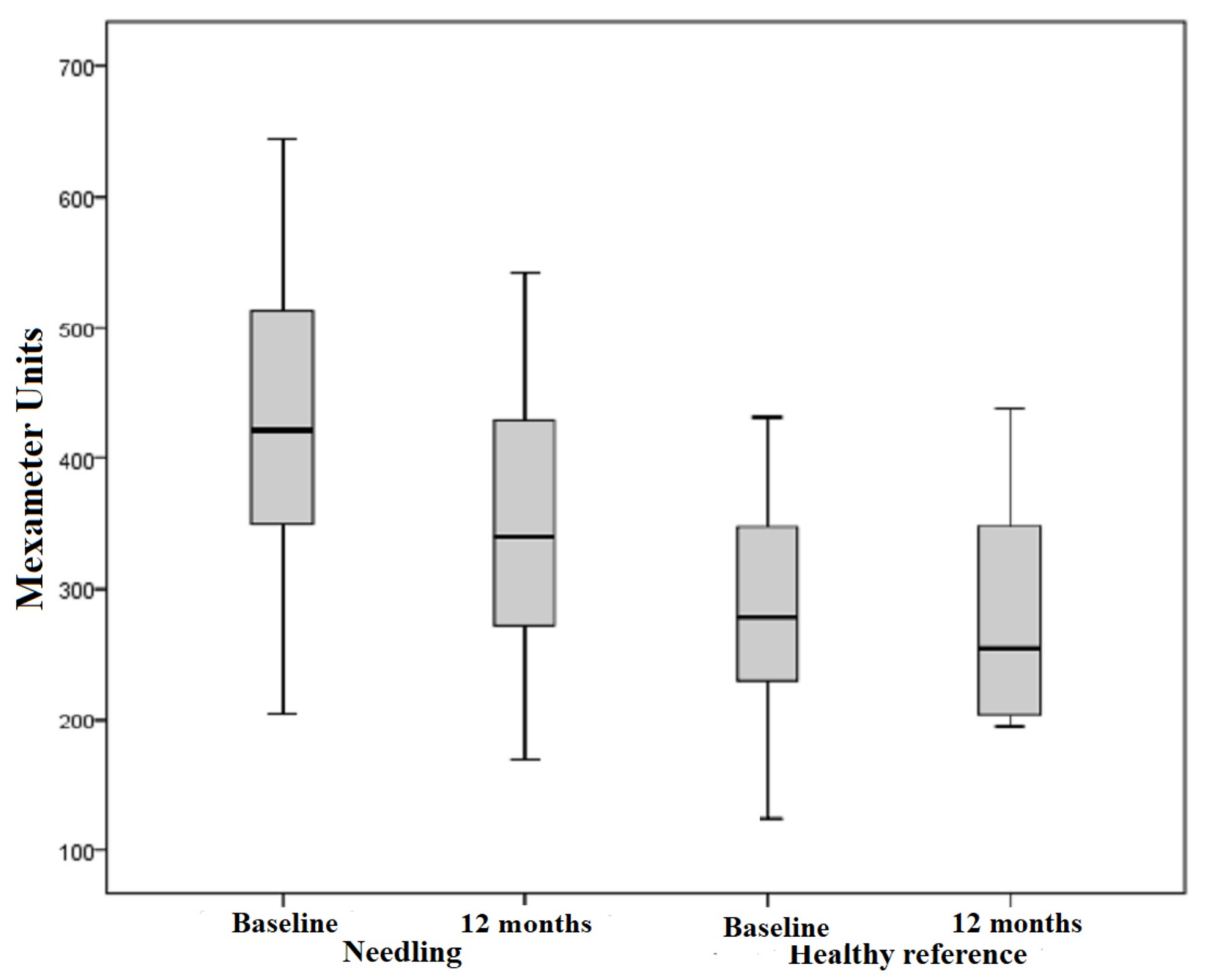
Erythema, pathological scars treated by medical needling and healthy skin pre- and one year postoperatively Box displays lower and upper quartiles, median value is indicated by the horizontal line, endpoints of upper and lower whisker display minimum and maximum values

**Figure 8:**
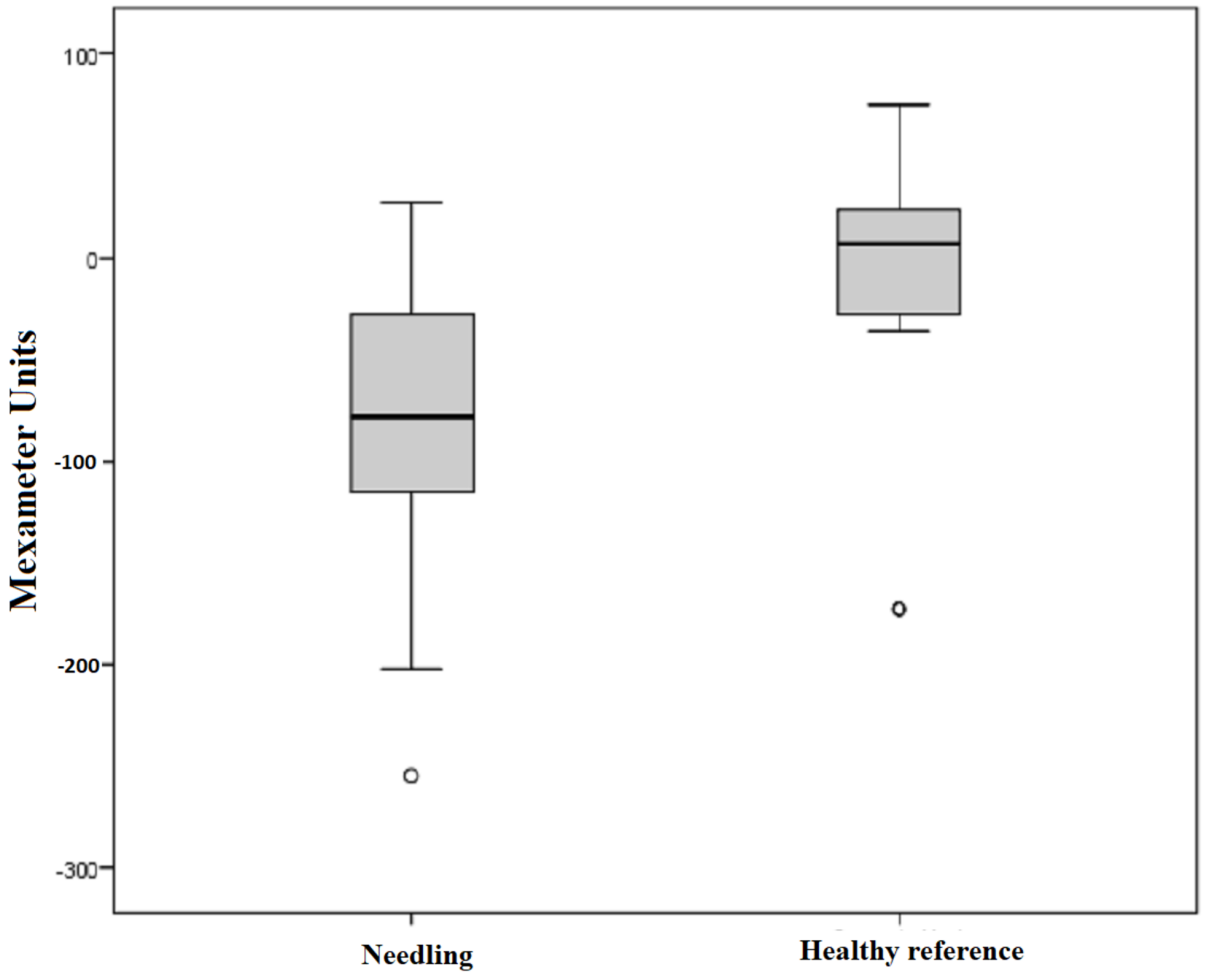
Difference in the erythema index for treated scars and healthy skin preoperatively and one year later Box displays lower and upper quartiles, median value is indicated by the horizontal line, endpoints of the upper and lower whisker display minimum and maximum values.

Our subject collective implied 47 scars out of 17 patients with pathological values of measurable erythema. The range of values for pathologically reddened skin is defined by higher scores compared to the standard values of healthy skin. The mean erythema index was 421.36 points preoperatively and 339.89 points postoperatively. This outcome shows a significant improvement of 19%, which is manifested in a measurable reduction in skin redness in the affected area. Measurements of erythema in the healthy reference also proved a minimal reduction of 4%. However, this outcome is not considered statistically significant with p = 0.635 (Figure 7). The difference between the pre- and postoperative measurements of each scar treated by medical needling showed a mean decrease of 79.87 points post-interventionally. Statistical significance cannot be recorded with p < 0.001 (Figure 8).

**Figure 9:**
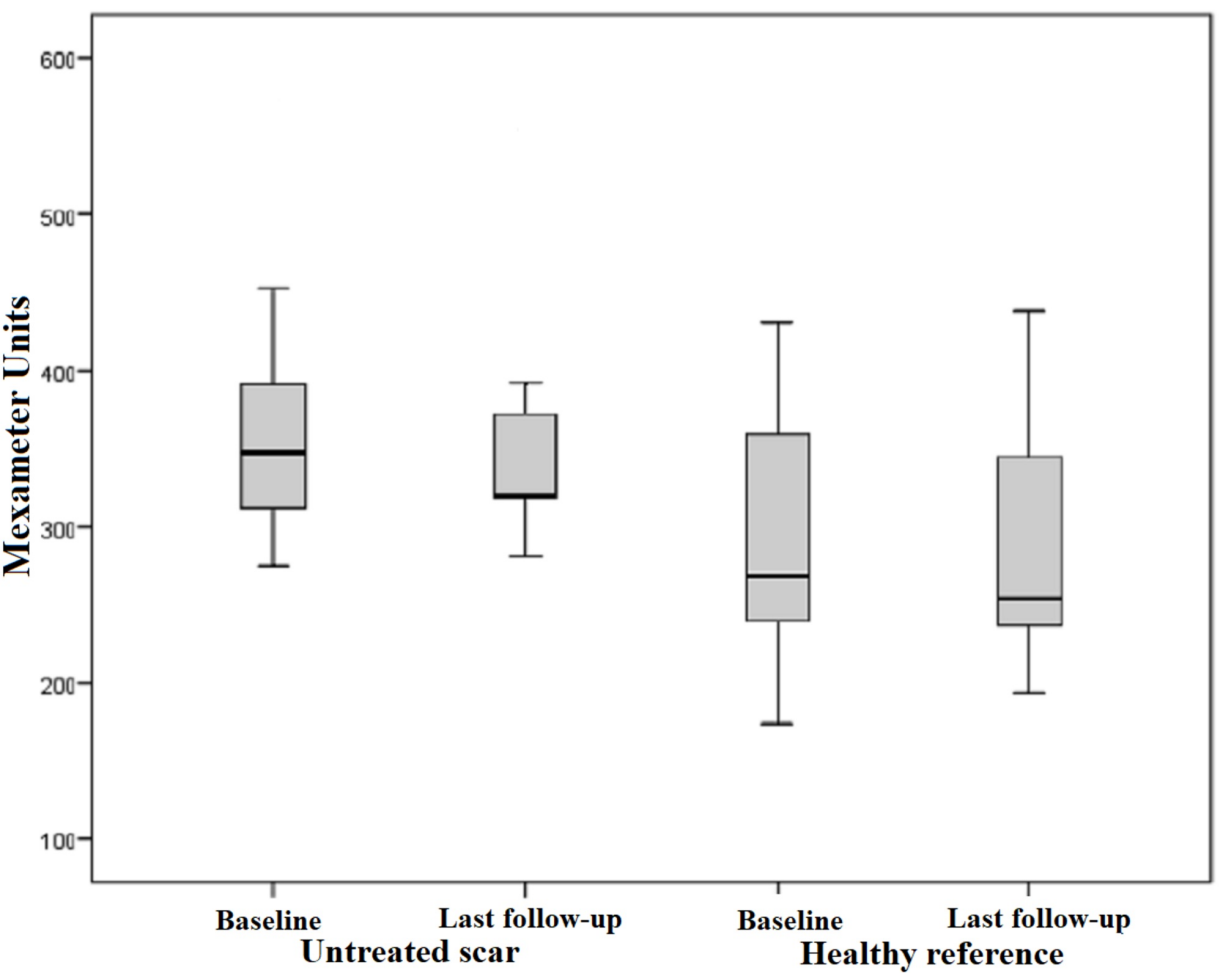
Erythema, untreated scars and healthy reference pre- and one year postoperatively Box displays lower and upper quartiles, median value is indicated by the horizontal line, endpoints of the upper and lower whisker display minimum and maximum values.

Less significant changes were evident when measuring untreated scars and healthy skin. Untreated scars showed an average redness of 368.09 points. After six months, a slight decrease to 358.18 points was apparent whereas an increase of up to 363.09 points could be observed at the final examination. This outcome is very similar to the initial measured value before needling. Comparing the pre- and postoperative measurements of the skin redness, a minimal reduction of 1% is notable. Thus, a statistical significance with p > 0.05 is not given. Furthermore, healthy skin remained more or less unchanged, as the preoperative erythema index of 289.,09 points increased to 289.45 postoperatively (Figure 9). According to that, a trend towards a significant difference could not be noticed when medical needling was not performed. Therefore, the minimum change of low relevance is evident in both untreated and healthy skin.

**Figure 10:**
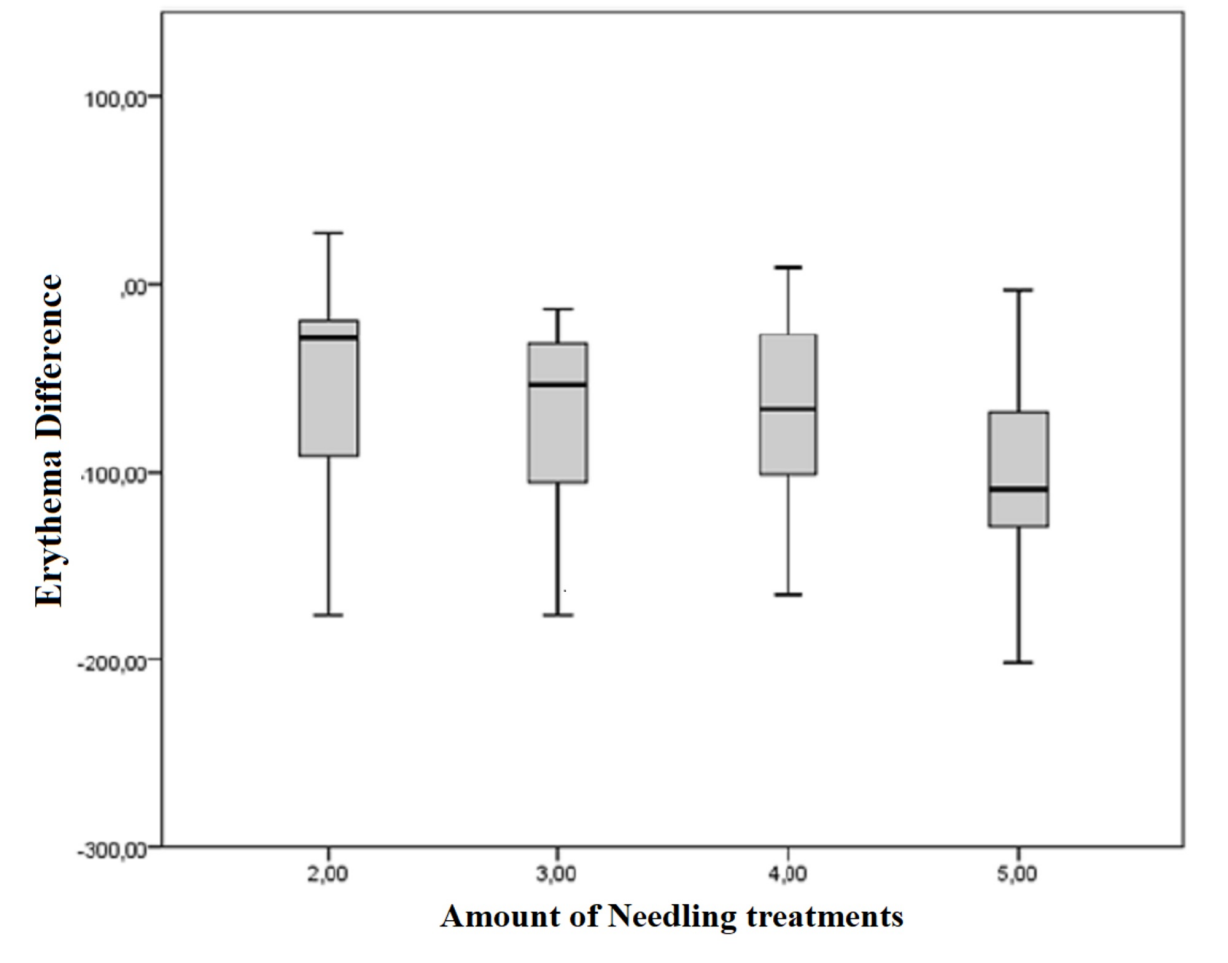
Difference of the pre- and postoperative measured erythema index dependent on the amount of treatments Box displays lower and upper quartiles, median value is indicated by the horizontal line, endpoints of the upper and lower whisker display minimum and maximum values.

Figure 10 depicts the difference between the pre- and postoperative measured erythema index in Mexameter units dependent on the number of treatments. The erythema index measured at the final examination after five treatments revealed an average reduction of 100.69 points. The postoperative median for the erythema index after the maximum number of treatments (five) was -108.5 points compared to -29 points after only two treatments. Indeed, this outcome represents an improvement in erythema due to repetitive needling. However, a linear regression of the pathologically increased redness of the skin cannot be proved (p = 0.081).

## Discussion

The diversity of treatment methods for the combination of hypertrophic and erythematous scars seems to be fairly limited when it comes to sustainable and long-term results. However, ablative treatments, such as surgical options, need to be replaced by less invasive methods and only used for emergency indications. Radical ablation is often followed by several side effects and risks for a potential degradation of the scar [12]. At best, poor and temporary improvement affects the scar situation with a short-term response whereas pathological erythema seems to be less improved but rather concealed. Compared to that, medical needling does not only preserve epidermal structures and their regenerative potential but also induces the synthesis of physiological collagen instead of scar collagen. The idea of PCI is realized by the method of medical needling, which reinforces the endogenous potential for regeneration. A modified wound healing cascade allows an increased expression of growth factors, such as the vascular endothelial growth factor (VEGF) and the tissue growth and transforming factor (TGF-ß). Both factors are also important for conventional wound healing with regard to angiogenesis and the differentiation of cells. Against this background, they also influence vascularization and the synthesis of structural proteins, such as collagen, in order to reduce the pathological appearance of persistent erythema. Medical needling stimulates gene expression and the proliferation of skin cells that are important for dermal remodeling. In this context, PCI changes the TGF-ß signal transduction pathway, as TGF-ß 3 reaches high levels of expression over the initial wound healing phase. This factor allows an association of medical needling with scarless wound healing [13-14]. The post-needling cascade causes the formation of a physiological “lattice-work“ collagen matrix of collagen type I instead of type III, which shows a parallel orientation and is less stable [15]. VEGF is an important factor that influences angiogenesis during the post-needling cascade. The controlled and limited release of VEGF creates new blood vessels in the dermis. The limited activity of VEGF for a desired period of time (post-inflammation) has the advantage of rapid wound healing due to a better perfusion of the treated area. This causes a temporary reddening of the affected skin area, which is not associated with the development of persistent erythema. The expression of specific proteins and the reorganization of the extracellular matrix affects the epidermal thickness [16]. The entire connective tissue framework appears thicker and denser after treating the scar with medical needling based on the proliferation of skin cells such as keratinocytes. Untreated hypertrophic burn scars tend to a thin and vulnerable epidermis, which is unstable and more transparent for local perfusion. This might support the appearance of persistent erythema through a thin skin texture. After needling, the intense blood circulation is less prominent and temporarily limited through a thickened epidermis. The scar color does not differ extensively from the surrounding healthy skin. The functional features of medical needling as a skin-normalizing treatment method create a homogenous image of initially reddened scars towards healthy skin.

In this context, we also figured out the importance of pre-treating the scar with highly dosed vitamin A and C products in combination with micro-needling, which is instructed before and after needling [17]. As a consequence, the maximal therapeutic outcome regarding epidermal thickness and a better prognosis for rapid healing could be stated. Nevertheless, medical needling has a positive effect on subcutaneous structures conveyed by VEGF as an important indicator of active angiogenesis. The expression of this growth factor creates new blood vessels and supports the proliferation of vascular endothelial cells [18]. This process happens after the initial inflammatory response of a vasoconstriction, which is followed by a massive vasodilatation in the wound healing phase. The effect on the vascular regulation explains the intense bleeding after needling as an important enhancer of healing progress. The better the blood perfusion of the skin tissue, the greater the bleeding, which accelerates the healing processes. Treatment with medical needling offers a preventative measure in order to avoid secondary wound healing that is a frequent cause of the development of hypertrophic erythematous scars. Scars that have healed by secondary intent do often occur in combination with other difficult issues such as infections [19]. With our study, we aimed at proving the effect of medical needling on hypertrophic burn scars with pathological persistent erythema. Based on the measurements with the Mexameter, scars treated by medical needling showed a measurable reduction of skin erythema after each treatment and especially after a year. On the contrary, we could not define significant changes regarding a pathologically increased skin redness in untreated scars. Similar to that, healthy skin has proven unchanged conditions of intact skin during the study. Two patients did not show a postoperative decrease of the erythema index. Due to the fact that other parameters have been positively affected by medical needling, they cannot be considered non-responders. An invalid performance of the procedure with an incorrect needling technique and low pressure might be a reason for a non-responder status. Improvements relative to the number of treatments are evident and depend on the individual itself. The patient cohort of this study is fairly limited to make concrete conclusions of a proportional relation. With POSAS, we were able to collect subjective, reliable data regarding erythema. Both, patient and observer, made very similar ratings of “overall opinion” and “redness” or “vascularization.” A tendency of pathological scarring towards healthy skin could be figured out for scars that have been treated by medical needling.

## Conclusions

Not only objective but also subjective evaluations of the examined parameter "erythema" display a coherent picture of a bilaterally affirmed improvement of the scar towards healthy skin. The efficient method of PCI improves epidermal structures biophysically and induces regenerative vascular processes within the post-needling cascade. Therefore, the release of several endogenous (growth)-factors (TGF-ß and VEGF) improves the scar quality aesthetically and functionally. Pathologically increased values of erythema reached standard values after repetitive treatments with medical needling. According to this, erythematous scars seem to be an indication for PCI, which offers a controlled and relatively simple method for the targeted treatment of persistent erythema.
